# Quality of life, stress and its coping strategies among the parents of children with autism spectrum disorder in Kathmandu Valley: A mixed method study

**DOI:** 10.1371/journal.pgph.0006997

**Published:** 2026-08-21

**Authors:** Sajani Basan, Amit Arjyal, Shital Bhandary

**Affiliations:** School of Public Health, Patan Academy of Health Sciences, Lalitpur, Nepal; ICDDR: International Centre for Diarrhoeal Disease Research Bangladesh, BANGLADESH

## Abstract

This study examines the quality of life, stress, and coping strategies of parents raising children with autism spectrum disorder in Kathmandu Valley. These parents face unique challenges, including caregiving demands, societal stigma, and limited support resources. Limited research exists on the psychosocial impacts these parents experience in Nepal, making this study crucial for understanding their struggles. This cross-sectional mixed-method study was conducted in autism care centers across Kathmandu Valley. Quantitative data from 140 parents was collected using validated scales (Autism Parenting Stress Index, Brief-COPE, Multidimensional Scale of Perceived Social Support, and Family Quality of Life Scale), analyzed with STATA/EZR, while qualitative data from semi-structured interviews was thematically analyzed using R package for Qualitative Data Analysis. Purposive sampling ensured diverse experiences, and the tools were pretested for reliability and validity. Parents’ greatest stressors were concerns about their child’s future independence (76.43%), acceptance (72.14%), communication (66.43%), and social development (51.44%). They primarily relied on active coping and planning, with family providing more consistent support than friends. Unemployed parents had a lower family quality of life than homemakers (p = 0.003). Greater use of emotion-focused (p = 0.017) and problem-focused coping (p < 0.001) was associated with lower family quality of life. Parents mostly faced stress over their child’s future and communication. They commonly used coping strategies like acceptance, emotional support, and religion, though some resorted to negative methods like self-blame, substance use and hitting the child. Challenges like societal stigma, limited support, financial strain, and disrupted family relationships were common. Parental support networks and perceived support significantly influenced family quality of life. This study highlights that these parents face various psychosocial burdens and struggles that affect their quality of life and they need interventions to reduce stigma, improve family well-being to ultimately improve their ability to care for their children.

## Introduction

Autism spectrum disorder (ASD) is a diverse group of neurodevelopmental disorders. ASD is distinguished by a certain level of difficulties in social communication and interaction. Atypical patterns of behavior and activity, such as trouble switching between activities, an attention to detail, repetitive behavior, and peculiar responses to sensations, are the additional traits of ASD [1–3]. The World Health Organization (WHO) reports that approximately 1 in 100 children have autism, and this prevalence appears to be increasing, likely due to greater awareness and improved diagnostic practices [2]. A recent review found the prevalence of ASD in Southeast Asia to be 6 per 1,000 children [4].

Caring for a child with autism spectrum disorder places significant demands on families, as they must manage both caregiving responsibilities and daily life. While raising any child is challenging, parents of children with ASD face more complex issues than those with typically developing children, making the situation particularly difficult [5,6]. Children with ASD often exhibit challenging behaviors and social difficulties that require intensive care [7,8]. Following the diagnosis of a child with ASD, parents face sudden and multifaceted challenges that impact their well-being and family quality of life. They are required to process complex information from specialists, consider various interventions, quickly accept the diagnosis, learn to interact with their child, and choose appropriate care programs [9–12]. The child’s characteristics such as symptom severity, communication difficulties, behavioral problems, and age further influence parental stress [13]. In addition, due to the lifelong care needs of children with ASD, parents also face a heavy caregiving burden.

In countries like Nepal, where awareness and support services for ASD are limited, these challenges become even more severe [14]. Caring for a child with ASD places heavy demands on parents, often resulting in physical and mental health problems, heightened stress and anxiety, social isolation, stigma, financial strain, and family disruption—all of which compromise the quality of life for both parents and the entire family [13,15–17]. Families frequently encounter social challenges such as exclusion, fear of judgment, and being perceived by society as having a “defective” child. These perceptions, combined with the intensive caregiving role, reduce opportunities for social interaction and intensify emotional and psychological distress [15,16]. Economic pressures, including loss of employment and the high costs of therapies and interventions, further compound the strain. Together, these social, psychological, and financial challenges create a substantial burden that contributes to stress, depression, and anxiety, ultimately diminishing parental quality of life [8,16,17].

ASD is also associated with significant stigma, which is influenced by public and professional perceptions of the condition and interpretations of observable autistic traits [18]. Because ASD primarily affects neurodevelopment rather than physical appearance, it can be considered a concealable stigma; children with ASD may not display visible characteristics, making their condition less apparent to others [19]. While children with ASD often face this type of stigma, their families are also affected through “courtesy stigma,” whereby family members experience discrimination or negative judgment due to their association with a person with ASD rather than any personal traits [20]. Consequently, both children with ASD and their parents may internalize stigma, leading to low self-esteem, heightened negative emotions, and social withdrawal. This internalization can further increase vulnerability to poor mental health and reduced overall well-being [21,22].

Research shows that parents of children with ASD experience higher stress and lower quality of life. A study in Oman found that the risks of depression and stress were 1.8 and 1.6 times higher in parents of children with ASD than in parents of typically developing children [23]. A study in Tamil Nadu, India found that a significant portion of parents of children with ASD (59.1%) had poor psychological quality of life, and 56.6% had poor physical quality of life. These parents also faced social isolation, stigma, and blame, which negatively impacted their overall health and well-being [14]. While raising a child with ASD, families often experience social withdrawal and this also results in personal and community challenges for the parents. The parents often experience stigma and discrimination within the society [17]. A study conducted among mothers raising children with ASD in Chitwan, Nepal showed that the parents faced a wide range of challenges like physical exhaustion, emotional issues like stress, denial, anxiety and anger, psychosocial burden, social isolation, societal ignorance and economic burden like job loss and expensive medical treatments [16].

Parents of children with ASD use different coping strategies to manage stress. While some adopt positive methods like seeking support and acceptance, others rely on harmful strategies like substance use and self-blame, which can worsen their well-being and quality of life [24,25]. The study conducted in Chitwan, Nepal showed that the parents coped using strategies like respite care, spiritual practices, positive thinking, and problem-focused strategies [16].

To cope with the stress and challenges of raising a child with ASD, parents commonly employ emotion-focused coping strategies, such as resting, expressing emotions in private or spiritual settings, and visiting places of worship. Some parents also use problem-focused coping strategies like understanding the nature of the child and putting more effort into taking care of the child [26].

Social support also plays a major role in the life of these parents. A study in India revealed a strong positive correlation between quality of life and perceived social support, and a negative correlation between parental stress and quality of life [27].

To support parents of children with ASD, it’s essential to create an environment that offers tailored information, resources, and practical help. Since each family faces unique challenges, strong support networks and evidence-based therapies can boost parents’ confidence and skills. Interventions should also involve autistic individuals and promote community efforts for greater inclusion and accessibility [2,28].

In Nepal, the rights and inclusion of persons with disabilities, including children with autism spectrum disorder, are recognized through several international commitments and national policies. Frameworks such as the Constitution of Nepal (2015), the Disability Rights Act (2017), and the Inclusive Education Policy for Persons with Disabilities promote equal rights, inclusive education, and social participation for individuals with disabilities. However, despite these policy commitments, the availability of specialized services, early diagnosis, parental counseling, and community-based support for families raising children with ASD remains limited in practice, leaving many parents to cope with significant psychosocial and financial challenges [16,29]. Awareness, diagnostic services, and structured support systems for ASD remain limited, particularly outside major urban centers. Although autism is recognized within the national disability framework, specialized services such as early diagnosis, behavioral therapy, parental counseling, and community-based support remain scarce and unevenly distributed.

Given the complex challenges faced by parents of children with ASD in Nepal, there is a critical need to assess their stress, coping strategies, and quality of life [16,30,31]. Limited research has been conducted on the psychosocial burden and mental health of Nepali parents raising children with ASD, making it essential to understand their unique challenges. Most studies have focused on quantitative assessments [30–32], but individual experiences vary based on social context and child severity, highlighting the need for qualitative research. Addressing this gap can help develop tailored interventions, reduce stigma, and strengthen support systems, ultimately improving the well-being of both parents and children with ASD.

So, this study aimed to assess the status of quality of life, stress, and its coping strategies among the parents of children with ASD in Kathmandu Valley.

## Materials and methods

### Study design

The study was a cross-sectional study with concurrent triangulation mixed method design conducted in the selected autism care centers of Bhaktapur, Lalitpur, and Kathmandu districts of Nepal.

### Study site

Kathmandu Valley was chosen as the study site because autism care centers are concentrated in these urban districts, and given the stigma faced by parents, selecting the valley made it easier to reach participants, as families from across the country visit these centers.

### Study participants

Parents of children below 18 years of age diagnosed with any subtype of ASD, who were reached through eight autism care centers and who provided informed written consent to participate in the study were included in the study.

### Inclusion and exclusion criteria

#### Inclusion criteria.

Parents of children below 18 years of age diagnosed with any subtype of ASD [33], who were reached through the selected autism care centers and who provided consent to participate in the study were included in the study.

#### Exclusion criteria.

Parents of children with ASD who could not be contacted through the selected autism care centers and those who did not provide consent for participation were excluded from the study.

### Sample size

A total of 140 parents of children with ASD were included in the quantitative component of the study. The sample size was calculated using G*Power 3.1.9.7 for multiple regression analysis, considering 12 predictor variables included in the study, using following formula:


$$\begin{array}{c} N=\frac{\lambda (\text{1}-R^{\text{2}})}{\rho_{Y(X_{j}*X_{\left(-j\right)})}^{\text{2}}}+p-\text{1} \end{array}$$

where,

N = Sample size

λ = non-centrality parameter

p = number of predictor variables

R = Population multiple correlation coefficient

f2 = Effect size of the parameter being tested

$\rho_{Y(X_{j}*X_{\left(-j\right)})\;}^{\text{2}}\;$= squared population semi-partial correlation coefficient

As no similar studies were available in Nepal or neighboring countries to estimate the population multiple correlation coefficient, conventional effect size estimates were used. According to Cohen (1988), effect sizes are categorized as small (0.02), medium (0.15), and large (0.35) [34]. In this study, a medium effect size (f² = 0.15) was assumed with 80% statistical power and a 5% level of significance. Based on these parameters, the minimum sample size calculated was 127. After adding a 10% non-response rate, the final sample size was 140, which was considered adequate for the multivariate analysis involving multiple predictors in the study.

For the qualitative component of the study, in-depth interviews were taken with the parents of the children diagnosed with ASD visiting the autism care centers till data saturation [35]. A total of 11 in-depth interviews were taken. The parents were first interviewed for the quantitative component and the suitable parents for in-depth interviews were identified through the response given by parents during quantitative interviews. While selecting the participants for the interviews, maximum variation was maintained. To maintain the maximum variation, interviews were taken with the participants varying in age, socio-economic status, and experiences related to raising children with ASD.

### Sampling technique

The study participants were selected purposively using a non-probability purposive sampling technique for both qualitative and quantitative components of the study. The children present in the selected autism care centers and their parents were identified and contacted with the help of the institutions. Parents were approached during therapy sessions at therapy centers and during pick-up and drop-off times at daycare centers. A purposive sampling method was used due to the difficulty in reaching and recruiting parents of children with ASD. This approach also enabled direct access to those most likely to provide relevant information. The centers were visited to discuss data collection from 24^th^ November, 2024 and recruitment of the study participants was started on 25^th^ November, 2024 and completed on 19^th^ January, 2025. In qualitative research, purposive sampling enables participants who are most relevant to the study aims to be identified and recruited, allowing for in-depth exploration of the experiences under investigation.

### Data collection tools and techniques

For quantitative data collection, a structured questionnaire in Nepali language was used to carry out face to face interviews with the parents (S1 Tool). The questionnaire included following parts:

Part 1: Socio-demographic characteristics of the study participants
Part 2: Parental stress using Autism Parenting Stress Index [36]. It consists of 13 questions related to three domains; core autism symptoms (stress related to the child’s social development, communication, closeness with the child, acceptance by others, and future independence), comorbid behaviors (stress associated with tantrums/meltdowns, aggressive behaviors, self-injurious behaviors, and difficulties with transitions) and comorbid physical issues (stress related to bowel problems, delayed toilet training, sleep problems, and diet/appetite issues). For each question, the total score ranges from 0 to 5 where 0 means “not stressful” and 5 means “so stressful sometimes we feel we can’t cope”. Higher the score, higher is the level of stress among the parents. It is an internationally validated tool which has been validated in several countries [37,38].
Part 3: Coping strategies adopted by the parents using Brief-COPE [39,40]. It is a 28 items likert scale where 14 subscales of coping are included. Each subscale has 2 questions and each question score ranges from 1 to 4, where 1 means the respondent does not use that coping strategy at all and 4 means the respondent uses that coping strategy a lot. Scores are presented for three coping styles; problem-focused coping, emotion-focused coping and avoidant coping.
Part 4: Perceived social support among the parents using Multidimensional Scale of Perceived Social Support [41]. It is a 12 items scale where score for each item ranges from 1 (very strongly disagree) to 7 (very strongly agree). The mean scores are calculated in 3 subscales; family, friends and significant other. Each subscale consists of 4 questions.
Part 5: Quality of life of the parents using Family Quality of Life scale [42–44]. It consists of 25 questions divided into 5 domains; family interaction, parenting, emotional wellbeing, physical/material wellbeing and disability-related support. Each question score can range from 1 (very dissatisfied) to 5 (very satisfied).

The validity and reliability of the quantitative tools used in this study are reported in the Validity and Reliability section that follows.

For qualitative data collection, an open-ended standardized interview guide in Nepali language was used to carry out in-depth interviews with the parents of children with ASD selected after quantitative interviews (S2 Tool).

### Validity and reliability

The validity and reliability of the quantitative tools were ensured through a rigorous process. The tools were developed based on literature review and expert guidance, with sociodemographic questions adapted from previous studies [5,30–32], NDHS 2022, and HMIS records. Forward and backward translation was conducted, and content and face validity were ensured through expert review and pretesting among 10 parents of children with ASD. Reliability was assessed during pretesting, and all four scales used in the study showed acceptable internal consistency, with Cronbach’s alpha values above 0.7.

The trustworthiness of the qualitative component was maintained through various strategies [45]. Credibility was ensured by developing the in-depth interview guide based on literature and expert input, using iterative questioning with probes, and conducting member checking. Dependability was addressed by calculating the Intercoder Percentage Agreement, which was found to be 70.37%, indicating acceptable reliability of coding. Transferability was supported by providing comprehensive background information and a detailed description of the research process. Confirmability was achieved by limiting the researcher’s role to that of a facilitator, avoiding personal bias, and allowing the data to be guided solely by participants’ responses. Member checking and reflexivity further strengthened the study’s confirmability.

### Data analysis

For quantitative data, STATA 13 and Easy R (EZR) software were used for analysis. Normality tests confirmed the data followed a normal distribution. Univariate analysis was conducted for sociodemographic variables (age of child, age of diagnosis, sex of child, age of parents, sex of parents, relation with the child, education level of parents, employment status of parents, occupation of parents, socioeconomic status of family, ethnicity and religion) and other independent variables (parental stress, coping strategies, perceived social support among the parents) and presented as frequency, percentage, mean, and standard deviation. Bivariate analysis was performed using simple linear regression. Variables with low frequency were recoded to ensure a reference category with a frequency of more than 30. Multiple linear regression was used to identify the independent effects of sociodemographic variables and other independent variables on family quality of life, with multicollinearity checked using Variance Inflation Factor (VIF). Final model assumptions and fit were also assessed.

For qualitative data, R Package for Qualitative Data Analysis (RQDA) software was used for coding. The in-depth interviews were audio-recorded, transcribed in Nepali, and then translated into English. Respondents were assigned unique codes to ensure anonymity. The translated data were imported into RQDA software (version 0.3-1) for coding, and codes were organized into categories, sub-themes, and themes using MS Excel. Braun and Clarke’s six-step thematic analysis was applied for further analysis to generate themes [46]. The findings were presented in terms of themes, sub-themes, and supporting verbatim excerpts. For the mixed-methods component, the qualitative and quantitative findings were triangulated and presented as converging, diverging, or expansive results.

### Ethical considerations

The study was approved by the Institutional Review Committee of PAHS (IRC-PAHS) (Reference: PHP2409201927). Permission was obtained from all selected autism care centers prior to data collection. Respondents were fully informed about the study through an information sheet in Nepali, and written consent was obtained. Participation was voluntary, and respondents had the right to withdraw at any time without explanation. Confidentiality was maintained by assigning respondent codes and avoiding the collection of identifying information. Interviews were conducted at the respondents’ convenience, ensuring no harm was caused. The data was used solely for academic purposes, and the researcher declared no conflict of interest.

## Results

### Quantitative results

#### Sociodemographic characteristics.

**Sociodemographic characteristics of children with ASD:** The median age at diagnosis was 2.5 years (IQR = 1), with half diagnosed by age 2 and 35% at age 3. The children’s median age at the time of the study was 4 years (IQR = 1), with the majority (55%) aged 0–4 years, followed by 40% aged 5–9 years. A small proportion were in the 10–14 (2.14%) and 15–18 (2.86%) age groups. Most of the children were male (78.57%), while females accounted for 21.43% of the sample.

**Sociodemographic characteristics of parents of children with ASD:** More than half of the parents were aged 31–40 years (52.86%) with a median age of 35 years. The majority of the parents were female (82.14%) and primarily mothers (76.43%), highlighting their role as primary caregivers. Over half had a bachelor’s degree or higher (55.71%), yet most were unemployed (62.14%), with homemaking being the most common occupation (60%). Most families belonged to the middle-income group, and household heads were generally well-educated, with many engaged in clerical or farming-related work. Socioeconomically, the majority were from the upper middle class (53.57%), with nearly equal distribution between joint and nuclear families. Ethnically, Brahmin/Chhetri made up the largest group (51.43%), and most respondents identified as Hindu (88.57%).

#### Status of stress, coping, perceived social support, and family quality of life.

Table 1 represents parental stress was assessed across three domains: core autism symptoms, co-morbid behaviors, and co-morbid physical issues. The highest mean stress score was observed in the core autism symptoms domain (mean = 11.46, SD = 5.44), indicating that parents experienced the most stress related to their child’s social development, communication, emotional closeness, societal acceptance, and future independence. Co-morbid behaviors had a moderate mean score (mean = 4.91, SD = 4.12), while co-morbid physical issues had the lowest (mean = 4.32, SD = 3.36), suggesting these factors were less stressful comparatively. The overall APSI score, reflecting total parenting stress, ranged from 1 to 50 with a mean of 20.7 (SD = 10.18).

**Table 1:** Scores on the Autism Parenting Stress Index domains (n = 140).

Domain	Minimum	Maximum	Mean	SD
Core autism symptoms	0	25	11.46	5.44
Co-morbid behaviors	0	18	4.907	4.12
Co-morbid physical issues	0	17	4.32	3.36
Total APSI scores	1	50	20.7	10.18

Table 2 highlights that the parents experienced the highest levels of stress due to core autism symptoms, particularly concerns about their child’s future independence (76.43%) and acceptance by others (72.14%), followed by difficulties in communication (66.43%) and social development (51.44%). Among co-morbid behaviors, tantrums and meltdowns (45%) and difficulty with transitions (37.14%) were the most commonly reported stressors. In the domain of co-morbid physical issues, toilet training delay (52.86%) and bowel problems (27.15%) were the primary sources of stress.

**Table 2:** Prevalence of stress among the parents (n = 140).

Items	Prevalence of stress^a^ (%)	Items	Prevalence of stress^a^ (%)
Your child’s social development	51.44	Your child’s diet	27.14
Your child’s ability to communicate	66.43	Bowel problems (diarrhea, constipation)	27.15
Tantrums/meltdowns	45.00	Potty training	52.86
Aggressive behavior (siblings, peers)	21.43	Not feeling close to your child	26.43
Self-injurious behavior	17.86	Concern for the future of your child being accepted by others	72.14
Difficulty making transitions from one activity to another	37.14	Concern for the future of your child living independently	76.43
Sleep problems	20.71		

^a^ *Prevalence of stress among the parents was determined by the percentage of responses at “Often Creates Stress” or higher in each item in the scale.*

Table 3 presents the mean scores of coping strategies used by parents of children with ASD, as measured by the Brief-COPE. Problem-focused coping was the most frequently used strategy (mean = 3.26, SD = 0.511), with active coping (mean = 3.47) and planning (mean = 3.41) being the most common approaches. Emotion-focused strategies were also utilized, especially acceptance (mean = 3.514), followed by positive reframing (mean = 3.185) and emotional support (mean = 2.871). Avoidant coping strategies were used less often, with self-distraction (mean = 2.514) being moderately employed, while denial (mean = 1.489), behavioral disengagement (mean = 1.375), and substance use (mean = 1.05) had notably low usage.

**Table 3:** Mean scores on the Brief-COPE domains (n = 140).

Sub-scales	Minimum	Maximum	Mean	SD
**Problem-focused coping**	1.88	4	3.26	0.511
Active Coping	1.5	4	3.47	0.696
Use of informational support	1	4	2.975	0.764
Positive reframing	1.5	4	3.185	0.66
Planning	1.5	4	3.410	0.641
**Emotion-focused coping**	1.33	3.42	2.439	0.426
Emotional support	1	4	2.871	0.816
Venting	1	4	2.457	0.836
Humour	1	3	1.114	0.351
Acceptance	1.5	4	3.514	0.565
Self-blame	1	4	1.992	1.016
Religion	1	4	2.689	1.011
**Avoidant coping**	1	2.75	1.607	0.416
Self-distraction	1	4	2.514	0.983
Substance use	1	2	1.05	0.210
Denial	1	4	1.489	0.695
Behavioral disengagement	1	4	1.375	0.784

Table 4 illustrates the levels of perceived social support among parents of children with autism spectrum disorder across three sources: family, friends, and significant others. The majority of parents reported moderate to high levels of support overall, with notable variations by source. Support from significant others was the highest, with 76.43% of parents reporting high support, underscoring the important role of close partners. Family support was also strong, with 73.57% reporting high support and only 12.14% indicating low support. In contrast, support from friends was more variable—only 47.14% reported high support, while 14.29% experienced low support, suggesting friends were a less consistent support source. Overall, 62.86% of parents experienced high levels of total perceived social support.

**Table 4:** Perceived social support among the parents (n = 140).

Sub-scales	Low support N (%)	Moderate support N (%)	High support N (%)
Family	17 (12.14)	20 (14.29)	103 (73.57)
Friends	20 (14.29)	54 (38.57)	66 (47.14)
Significant other	10 (7.14)	23 (16.43)	107 (76.43)
Total scores	5 (3.57)	47 (33.57)	88 (62.86)

Table 5 reveals that parents of children with autism spectrum disorder reported an overall good family quality of life, with a mean score of 3.911 (SD = 0.675). The highest satisfaction was observed in the Physical/Material Well-being domain, followed closely by Family Interaction, indicating that most families had stable living conditions and positive relationships. Moderate satisfaction was reported in the areas of Disability-related Support and Parenting, though variations suggested some families faced challenges in accessing resources and managing parenting responsibilities. Emotional Well-being had the lowest mean score, highlighting that emotional stress and psychological struggles were common.

**Table 5:** Mean scores on the domains of Beach Center Family Quality of Life Scale (n = 140).

Domains	Minimum	Maximum	Mean	SD
Family interaction	1	5	4.092	0.810
Parenting	1.5	5	3.803	0.841
Emotional Wellbeing	1	5	3.61	0.800
Physical/Material Wellbeing	1	5	4.107	0.625
Disability-related support	1.5	5	3.85	0.89
Total FQOL scores	1.52	5	3.911	0.675

#### Correlation between family quality of life and independent variables.

Table 6 revealed that parental stress, especially related to autism symptoms and co-morbid behaviors, negatively impacted family quality of life (FQOL). Coping strategies like emotion-focused and problem-focused coping were also associated with lower FQOL scores. In contrast, perceived social support, especially from family and significant others, showed a strong positive relationship with FQOL.

**Table 6:** Correlation between family quality of life and independent variables.

	Family Quality of Life	
	r	p-value
**Parental stress**		
Core autism symptoms	-0.75	**p < 0.001**
Co-morbid behaviors	-0.763	**p < 0.001**
Co-morbid physical issues	-0.584	**p < 0.001**
Total APSI score	-0.903	**p < 0.001**
**Coping strategies**		
Avoidant coping	-0.113	0.183
Emotion-focused coping	-0.563	**p < 0.001**
Problem-focused coping	-0.845	**p < 0.001**
**Perceived social support**		
Family	0.901	**p < 0.001**
Friends	0.0556	0.514
Significant other	0.544	**p < 0.001**
Total MSPSS scores	0.685	**p < 0.001**

#### Linear regression analysis.

Table 7 shows that several factors were significantly associated with family quality of life among parents of children with ASD. In unadjusted models, unemployed parents and those whose household heads were semi-professionals, skilled workers or unemployed reported lower family quality of life. Greater perceived social support was associated with better outcomes, and higher use of emotion-focused and problem-focused coping strategies was related to lower family quality of life.

**Table 7:** Simple and multiple linear regression analysis between family quality of life and independent variables.

Variables		Unadjusted Models			Adjusted Model		
		β	p-value	R^2^	β	p-value	R^2^
**Age of Respondent**			0.1112 (Wald)				0.7619
	21-30 years	Ref		0.03107	Ref		
	31-40 years	0.0514	0.7118		0.0336	0.668	
	> 40 years	0.3133	0.0597		0.0113	0.909	
**Employment status of respondent**							
	Employed	Ref			Ref		
	Unemployed	-0.1518	0.198	0.01196	0.0626	0.462	
**Occupation of respondent**			**0.0024 (Wald)** ^**b**^				
	Agriculture	-1.1033	0.0925	0.1085	-0.6434	0.097	
	Homemaker	Ref			Ref		
	Job/Service	0.1438	0.3104		0.04527	0.657	
	Labour	–	–		–	–	
	Business/commerce	0.0566	0.7013		–	–	
	Unemployed	-1.5833	0.00083		-0.8384	**0.003** ^**b**^	
	Others	–	–		–	–	
**Main occupation of the household head**			**0.0273 (Wald)** ^**b**^				
	Profession	-0.3052	0.0718	0.08599	-0.0039	0.968	
	Semi profession	-0.5572	0.0139		-0.0414	0.750	
	Clerical, shop owner, farmer	Ref			Ref		
	Skilled worker	-0.3872	0.0368		-0.0259	0.809	
	Semi-skilled worker	-0.0572	0.8071		-0.1067	0.412	
	Unskilled worker	–	–		–	–	
	Unemployment	-0.3792	0.0258		-0.0375	0.733	
**Family Type**							
	Nuclear	Ref			Ref		
	Joint	-0.1351	0.238	0.01006	0.0197	0.771	
**Ethnicity**			0.0611 (Wald)				
	Dalit	-1.00	0.1387	0.05136	-0.1096	0.775	
	Janajati	Ref			Ref		
	Madhesi	-0.1415	0.4922		-0.1293	0.279	
	Muslim	–	–		–	–	
	Brahmin/Chhetri	-0.2877	0.0176		-0.0712	0.303	
	Others	–	–		–	–	
**Coping Strategies**							
	Avoidant	-0.1836	0.183	0.01281	0.0286	0.713	
	Emotion-focused	-0.8926	**p < 0.001**	0.317	-0.2217	**0.017** ^**b**^	
	Problem-focused	-1.1162	**p < 0.001**	0.7134	-0.8981	**p < 0.001**	
**Perceived social support**		0.4216	**p < 0.001**	0.4696	0.0426	0.355	

^b^*denotes p-value<0.05*.

In the multivariate analysis, independent variables identified as significant in the bivariate analysis or those with a Wald’s p-value or p-value less than 0.25 were included. The model’s ANOVA was statistically significant (F-statistic = 20.21, p < 0.001), confirming the model’s validity. The coefficient of determination (R² = 0.7619) indicated that 76% of the variance in family quality of life could be explained by the independent variables. Significant predictors of family quality of life included the occupation of parents, with unemployed parents reporting lower quality of life compared to homemakers (p = 0.003), and both higher emotion-focused (p = 0.017) and problem-focused (p < 0.001) coping strategies being associated with lower family quality of life.

### Qualitative results

#### Themes identified in qualitative study.

All 11 parents interviewed in qualitative study were mothers. The age of the mothers ranged from 29 to 53 years. All of them had some level of formal education, from secondary level to bachelors and above. Most of the mothers were homemakers and some were employed in formal sector. Some mothers had left their job and become homemakers after their children were diagnosed with ASD.

Seven broad themes were identified from the thematic analysis of the qualitative data.

#### Social and institutional perceptions of autism.

This theme explores parents’ perspectives on autism spectrum disorder (ASD), including their understanding, societal attitudes, stigma, and community acceptance. Many parents felt that society lacks awareness of ASD, often viewing it as a communicable disease or a result of bad luck. Some parents were also unaware of ASD and attributed it to factors like isolation during COVID-19 or excessive screen time. Acceptance emerged as a major challenge—families, schools, and communities often rejected children with ASD. Even parents struggled to accept the diagnosis. Misconceptions and stigma were widespread, with many parents facing blame, criticism, and social exclusion, even from their own families.

*“The thing is, as soon as people hear the word “autism,” they think it is a contagious disease. …. They don’t even let their children play outside with my child. They think that if their children see my child’s actions, they will start copying him. They hold that kind of negative thinking.”*                               -*IDI 02*

*“One school even said they wouldn’t accept my child. They said, “We can’t take the child, we won’t be able to handle him.” That’s how it usually is. Most schools say, “We can’t.”*                               -*IDI 04*

*“At first, when I talked about autism, they didn’t believe it. They said things like, “It’s because of bad luck or something supernatural.”*                               -*IDI 11*

#### Challenges faced by the parents of children with ASD.

This theme highlights the challenges faced by parents of children with ASD, including personal, financial, behavioral, and caregiving difficulties. Many experienced emotional distresses after the diagnosis, including stress, anxiety, and anger. Care demands impacted their physical health, causing exhaustion and sleep loss. Some left their jobs or struggled to balance work and family, affecting careers and interests. Financial strain was common due to therapy and care costs. A major challenge was managing behaviors like aggression, self-harm, sleep issues, tantrums, and eating difficulties. Taking children out in public was also difficult for many parents.

*“It has affected me a lot. Lack of sleep increases my diabetes. I had postnatal diabetes, which started when I was pregnant with my daughter. …. And when my daughter doesn’t sleep at night, and I have to stay awake with her, my fasting blood sugar and everything else stays high. I am on medication for it.”*                               -*IDI 10*

*“I don’t have any time for that. My career is completely on hold for now… I can put my career on hold for a few years and restart later, but if my child’s development is delayed now, it will affect him for life. So, for now, my entire focus is on him.”*                               -*IDI 09*

*“If he gets angry, he might pinch someone, pull their hair, or grab them tightly. Earlier, he also used to bite a little.”*                               -*IDI 05*

#### Parental stress.

This theme explores the various types of stress experienced by parents of children with ASD, including stress from the child’s behavior, concerns about the future, and societal or familial attitudes. Most parents reported stress due to behaviors like aggression, tantrums, poor communication, and self-harm. A major source of stress was fear about the child’s future, especially regarding independence and societal acceptance after the parents are gone. Additionally, negative reactions and perceptions from family and society further contributed to their stress.

*“There are moments when I don’t understand what he wants. He tries to say one thing, but I understand something else. Neither this nor that works, and since I don’t understand him, calming him down becomes very difficult. In those moments, I feel overwhelmed—I feel like crying.”*                               -*IDI 09*

*“My biggest fear is about the future. What will happen tomorrow? Right now, things are fine, but how long can I continue like this? If I have to do this until the very end, how will I manage? What will happen when I am not here anymore? As long as I am here, I can take care of him. But after me, what will happen? That is a major concern.”*                               -*IDI 05*

#### Coping strategies adopted by parents.

This theme explores how parents cope with the stress of raising a child with ASD. While few used avoidant strategies like denial or withdrawal, many relied on emotion-focused coping, such as seeking emotional and religious support. Others used problem-focused approaches like planning, attending trainings, and sharing experiences with other parents.

*“I don’t feel like going anywhere now. Attending parties or weddings feels uncomfortable because of that.”*                               -*IDI 01*

*“I took PCTP (Parent-Child Training Program) training within a week of learning about my child’s autism. After that, I learned a lot of things, and slowly, everything started getting better.”*                               -*IDI 02*

*“I have received emotional support. My family is educated. My in-laws are no longer alive, but when they were, they were very understanding. Even from my husband’s side, I never faced any accusations or blame. Everyone in the family has loved my child.”*                               -*IDI 10*

*“I even changed my religion from Hindu to Christian for my son. After that, there were improvements in my son’s health. His health started to be good.”*                               -*IDI 02*

#### Community and family support in autism care.

This theme explores the social support parents perceive, including help from family, friends, and society for both themselves and their child. While many parents felt supported by their families and were satisfied, others expressed dissatisfaction due to a lack of help. Some also reported receiving strong support from friends, neighbors, and the wider community.

*“I consider myself lucky because my husband supports me, and my family supports me. Not everyone has that.”*                               -*IDI 10*

*“No one is supportive. Even my husband doesn’t say anything when people criticize me. My mother-in-law and sister-in-law always get angry at me and treat me badly.”*                               -*IDI 11*

*“I have received support, especially at work. When I told them about my son’s condition, they were understanding. I can leave the office early or arrive a bit late—by about half an hour. So, I have received support there as well.”*                               -*IDI 05*

#### Impact of autism diagnosis on family’s quality of life.

This theme explores how an ASD diagnosis affects family quality of life, including relationships, emotional and material needs, and caregiving support. While some parents said family dynamics remained stable, others reported strained relationships. Many received strong support in raising and teaching their child, though some felt unsupported. Most parents were satisfied with their family’s ability to meet material needs, but a few expressed dissatisfactions.

*“But between my husband and me, it had a significant impact. …. When I was frustrated, I would take it out on my husband, and he would do the same to me. He would come home at the time when I am angry, say something, and I would get irritated. So, my frustration would be directed at him. This caused strain in our relationship.”*                               -*IDI 10*

*“Wherever I take him, whatever I do, it’s all on me. I have no hope that someone else will take care of him.”*                               -*IDI 11*

*“Financially, it is hard for Nepali people to manage while staying in Nepal. Therapy, school, and his diet, his things, toys, taking him outside, and everything cost a lot. Since our earnings in Nepal were not enough, my husband had to go abroad.”*                               -*IDI 09*

#### Empowering parents: Management, programs, and personal actions.

This theme highlights parents’ views on what can empower them to better care for their children with ASD. It includes personal efforts they believe are helpful, as well as suggestions for programs and initiatives to support parents. Based on their experiences, they shared actions other parents can take and recommended measures needed in Nepal to support families of children with ASD.

*“The first thing we need to do is educate ourselves. We need to be mentally prepared that our child has autism. It’s okay, this is a challenge, and I will take on this challenge. Now, how do I teach them? How do I create milestones for the things they can’t do? What things are they unable to do at their age, and how can I master them for them? I need to create goals for them and target them, and then we’ll achieve those goals.”*                               -*IDI 10*

*“It would be beneficial if parents could gather occasionally to discuss challenges, ways to move forward, and what works for different children. If parents shared their experiences with each other, it would give everyone new ideas.”*                               -*IDI 08*

*“Anyone who has a connection with policymakers or is in a position to talk to them should advocate for a policy to be introduced in our country that provides support, like what is done for other disabilities. …. The government needs to introduce policies, provide allowances, free medications, build schools, or whatever is necessary for our children.”*                               -*IDI 10*

### Mixed method results

#### Triangulation of quantitative and qualitative findings.

Through data triangulation, the data from quantitative and qualitative studies was used to identify the convergent, divergent and expansive findings.

#### Convergent findings.

**Parental stress about core autism symptoms:** Survey results showed the highest stress in the domain of core autism symptoms (Mean = 11.46, SD = 5.44). Parents were most concerned about their child’s future independence (76%), acceptance by others (72%), communication (66%), and social development (51%). Qualitative interviews confirmed these concerns, with parents frequently expressing worry about communication and the child’s uncertain future.

“*The main thing is that he can’t speak. He speaks only a few words. The fact that he doesn’t talk causes me a lot of stress.*” – IDI 08

“*Mainly about the future… will others accept him? What will happen as he grows up? There will obviously be some stress*.” – IDI 04

**Parental stress about co-morbid physical issues:** Stress related to physical issues was lowest (Mean = 4.32, SD = 3.36). Most parents were not troubled by bowel, diet, or sleep problems, but many found toileting difficult (53%). Interviews reflected the same.

“*The only thing that stresses me a bit is that when he goes to the toilet, he completely takes off all his clothes. I wish I could change that habit—it would make things a little easier.*” – IDI 07

**Coping strategies:** Problem-focused coping was most common (Mean = 3.26, SD = 0.51), with parents actively planning, seeking information, and reframing situations positively. Qualitative findings supported this.

“*Being sad won’t cure autism. I have to forget my sorrow, stay motivated, and move forward. The happier I am, the stronger I will be—and being strong will benefit my child as well*.” – IDI 10

Emotion-focused coping (Mean = 2.44, SD = 0.43) included acceptance, emotional support, venting, and religious practices. Nearly all parents had accepted the diagnosis.

“*I even changed my religion from Hindu to Christian for my son. After that, there were improvements in my son’s health.”* – IDI 02

Avoidant coping had the lowest scores (Mean = 1.61, SD = 0.42). Parents sometimes used self-distraction, but none reported substance use or denial.

“*When we start feeling exhausted, we find ways to refresh ourselves… If our daughter doesn’t want to go out, at least we go out with friends for a break.”* – IDI 10

**Social support:** Support was strongest from spouses (Mean = 5.84, SD = 1.28), followed by family and friends. Interviews confirmed that spousal support was central.

“*I consider myself lucky because my husband supports me, and my family supports me. Not everyone has that.”* – IDI 10

**Family quality of life (FQoL):** Physical/material wellbeing scored highest (Mean = 4.11, SD = 0.63), while emotional wellbeing was lowest (Mean = 3.61, SD = 0.80). Parents often reported financial manageability but emotional strain, including giving up careers.

“*It’s manageable. We don’t have external financial support, but somehow, we manage.*” – IDI 06

“*My career is completely on hold for now… if my child’s development is delayed now, it will affect him for life.”* – IDI 09

**Education of parents and family quality of life:** Education level of parents had no significant relation with FQoL; instead, training and experience shaped outcomes.

“*I also took some training… and after that, I gained some knowledge about how to manage it.”* – IDI 05

**Parental stress and family quality of life:** A strong negative correlation was found between parental stress and FQoL (r = –0.90, p < 0.001). Parents with higher stress reported poorer family functioning.

“*The stress caused conflicts within the family… There was tension from every side.”* – IDI 10

#### Divergent findings.

**Occupation of parent and family quality of life:** In the qualitative study, employed parents reported better family quality of life due to greater emotional and material support, while homemakers faced heavier workloads and poorer wellbeing. However, quantitative analysis showed no significant difference between the two groups.

*“They do support me in raising him. For example, they pick him up from school, feed him, and help with other tasks.”* -IDI 08

This divergence may be explained by differences in how occupation influences day-to-day experiences versus overall measurable family quality of life. While qualitative findings suggested that employed parents benefited from greater emotional and practical support (e.g., shared caregiving responsibilities at home and workplace flexibility), these advantages may not be substantial or consistent enough to produce statistically significant differences in overall FQoL. Additionally, both employed and homemaker parents continue to face shared structural challenges such as caregiving burden and financial or emotional stress, which may minimize observable differences between the groups in quantitative analysis.

**Main occupation of household head and Family Quality of Life:** In the quantitative study, household head’s occupation was significantly related to family quality of life, with semi-professional households reporting lower scores than clerical, shop owner, or farmer households. However, qualitative findings did not identify occupation as an influencing factor. The divergence may be explained by the fact that occupation-related differences emerged as a statistical pattern in the quantitative data but were not readily perceived or emphasized by participants during interviews. Qualitative narratives tended to focus more on caregiving burden, stigma, and service limitations rather than household occupational status, which may be an indirect and less salient determinant of family quality of life in daily lived experiences

**Family type and family quality of life:** Qualitative findings showed that parents in nuclear families had better family quality of life, while those in joint families reported dissatisfaction with in-law support. Quantitative results, however, found no significant association between family type and quality of life. Although qualitative findings suggested that nuclear families experienced better support dynamics and that some parents in joint families reported dissatisfaction with in-law support, these differences may not have been strong or consistent enough across the sample to reach statistical significance. Additionally, variability in family functioning within both nuclear and joint families may have reduced observable differences in overall family quality of life in the quantitative analysis

**Income status of family and family quality of life:** Qualitative interviews indicated that higher-income families had better quality of life, as they could afford therapies and trainings. However, quantitative analysis showed no significant association between income and family quality of life.

*“Right now, I’m not able to provide additional therapy. I couldn’t afford it—it’s quite expensive. That’s one of the reasons I enrolled him in a special school, so that he can get therapy there as well. At least, having him involved in the school helps with that.”* -IDI 05

The divergence may be explained by the fact that, while qualitative findings emphasized higher income as enabling better access to therapies and support services, this advantage may not have been consistent or strong enough across participants to yield a significant quantitative association. Moreover, similar levels of caregiving burden and emotional stress across income groups may have reduced detectable differences in overall family quality of life.

**Social support and family quality of life:** Qualitative findings showed that strong spousal, family, and community support improved parents’ quality of life, but quantitative analysis found no significant association between perceived social support and family quality of life. *“I have received support, especially at work. When I told them about my son’s condition, they were understanding. I can leave the office early or arrive a bit late—by about half an hour. So, I have received support there as well*.” -IDI 05

This divergence may be explained by the informal and context-specific nature of social support, which, although perceived as helpful in daily caregiving, may not be sufficient in intensity or consistency to influence overall family quality of life.

**Coping and family quality of life:** Quantitative results showed both emotion- and problem-focused coping were negatively associated with FQoL, a finding not evident in qualitative narratives. The divergence between quantitative and qualitative findings may be explained by the contextual realities highlighted in the qualitative data. Although parents reported using both problem- and emotion-focused coping strategies, in-depth interviews revealed persistent exhaustion, social isolation, stigma, and limited structural support. These conditions suggest that coping efforts often function as short-term survival mechanisms rather than strategies that effectively improve overall family quality of life. In this context, structural constraints may outweigh the potential benefits of coping, helping to explain why higher use of coping strategies was associated with lower FQoL in the quantitative analysis.

#### Expansive findings.

Qualitative interviews uncovered additional themes not explored quantitatively.

**Marital relationships:** Qualitative interviews revealed that a child’s ASD diagnosis sometimes strained marital relationships, with some mothers reporting blame from husbands and fathers’ denial of the condition.

“*Taking care of my daughter… made me upset. When I was frustrated, I would take it out on my husband, and he would do the same. This caused strain in our relationship.*” – IDI 10

**Perceived causes of ASD:** Qualitative interviews showed that many parents believed ASD was caused by isolation, especially among children born during COVID or frequently using mobile phones.

“*I wonder if it was because I let him watch too much on the phone when he was little.*” – IDI 04

**Acceptance in schools and community:** Qualitative interviews revealed that children with ASD faced rejection in schools and the community, including school expulsion and exclusion from play, creating additional stress for parents.

“*The teacher even spoke harshly, saying, ‘Your child has an intellectual disability; you should enroll him in another school*.’” – IDI 03

**Stigma and misconceptions:** Qualitative findings indicated widespread misconceptions and stigma about ASD, with some viewing it as a mental illness, bad fate, or contagious, leading to social exclusion.

“*They said things like, ‘It’s because of bad luck or something supernatural*.’” – IDI 11

**Parental hesitation to socialize:** Qualitative interviews showed that parents often hesitated to socialize due to stigma, fearing misunderstanding and lack of acceptance from others.

“*Our society doesn’t accept it… At first, they say, “It’s fine,” but later, they gossip behind our backs—that’s the kind of society we have. That’s why, when it comes to relatives, even if we go, I don’t feel comfortable enough to fully open up*.” – IDI 04

**Coping through hitting the child:** Qualitative findings revealed that some parents physically disciplined their child with ASD to manage stress and anger, especially during tantrums or aggressive behavior.

“*Sometimes, I even hit him. When he misbehaves and makes me angry, I feel like hitting him*.” – IDI 11

**Parent networks:** Qualitative interviews highlighted parents’ networks as a coping resource, where sharing experiences and support in parent groups helped manage stress and improve child care.

“*I also have been chatting in the autism-specific group… from them, I get a lot of encouragement.”* – IDI 01

**Parental relocation to the valley for child’s therapy:** Several parents relocated to the Kathmandu Valley, residing in rented accommodations to access therapy and specialized services for their children with ASD, given the limited availability of such services outside the valley.

“The doctor said, “There are no therapy centers here. He needs therapy. In Kathmandu, there is Nepal Autism Care. You can take him there.” But he didn’t specify the address. So, I just noted down the name and searched about it in Google after coming back to home.” – IDI 03

**Health service–related factors:** During data collection, respondents expressed dissatisfaction with the services provided by counselors and therapists. Some parents reported seeking services at centers employing therapists from India, citing the perceived higher quality of care. This was also confirmed by the manager of one such center.

Overall, quantitative and qualitative findings converged on core stressors, coping patterns, and the inverse link between stress and family quality of life. Divergences highlighted differences in how family characteristics relate to wellbeing. Expansive findings revealed broader social and cultural dimensions like stigma, school exclusion, marital strain, and parent networks that extend beyond the quantitative framework.

## Discussion

Our study found that the maximum mean stress score was 11.46 with a standard deviation of 5.44 in the domain “core autism symptoms”. The mean stress score was 4.907 in the domain “co-morbid behaviors” and 4.32 in co-morbid physical issues. Similar results were shown by a study in Greece which reported that the highest stress was experienced in the domain of “core autism symptoms” (Mean = 10.92, SD = 4.11), followed by stress related to co-morbid behaviors (Mean = 4.98, SD = 3.80) and co-morbid physical issues (Mean = 4.30, SD = 3.36) [5]. In our study, the leading causes of parental stress were concerns about their child’s future—specifically, independent living (76.4%), acceptance by others (72.1%), communication (66.4%), and social development (51.4%). International studies show similar concerns but with varying prevalence levels. For example, stress prevalence was reported as 45.9% among caregivers of children with ASD in Oman [23], 55.4% in the Netherlands [47], 37.5% with clinically significant stress in Turkey [25], and 61% with mild to severe stress in Kazakhstan [48].

In Nepal, these concerns are magnified by cultural beliefs about disability and stigma, which often frame autism as a family burden or even as the result of past wrongdoing.

The qualitative findings of our study revealed that parents experienced significant stress mainly due to worries about their child’s future, especially in their absence. Additional stressors included challenges related to the child’s behavior, such as communication difficulties, social interaction issues, toileting problems, aggression, and self-harming behaviors. Family and societal pressures like stigma, misconceptions, and blame also contributed to parental stress. These findings are consistent with reports from Nepal [16] and Ethiopia [26], where stigma and blame aggravated parental stress. Thus, while stress is a universal feature of parenting a child with ASD, the Nepali context intensifies it through stigma, limited public awareness, and cultural pressures for children to fulfill expected social roles such as marriage, independence, and care for parents in old age.

In our study, parents of children with ASD primarily relied on problem-focused coping strategies, with a mean score of 3.26 (SD = 0.511), indicating a proactive approach to managing challenges. Among these, active coping was the most commonly used (mean = 3.47, SD = 0.696). In the emotion-focused category, acceptance was the most frequently used strategy (mean = 3.514, SD = 0.565), followed by positive reframing (mean = 3.185, SD = 0.66). Avoidant coping strategies were the least used, with some parents using self-distraction (mean = 2.514, SD = 0.983), while denial, substance use, and behavioral disengagement were rarely reported. Similar results were shown by a study conducted in Greece where parents most commonly used coping strategies like planning (mean/SD = 6.67/1,35), acceptance (mean/SD = 6.50/1.38), active coping (mean/SD = 5.92/1.66) and positive reframing (mean/SD = 5.71/1.79). The study also showed that avoidant coping was least common among the parents [5]. Several other international studies showed the results similar to our study. The studies found that parents mostly used problem focused and emotion focused coping strategies and avoidant coping strategies were very less used by the parents of children with ASD [26,49,50].

Qualitative insights revealed that Nepali parents often cope by using strategies like positive reframing, acceptance, engaging in religious activities and self-blame. Many sought emotional support within extended families and local parent networks, consistent with Nepal’s collectivist values. Similar patterns are documented in Chitwan [16,51] and South Africa [52], where religion, involvement in support groups and family solidarity serve as primary coping resources. These findings suggest that while coping strategies resemble those in other countries, the Nepali reliance on faith, collective family responsibility, and peer networks reflects cultural adaptations of coping mechanisms.

In our study, the majority of parents of children with autism spectrum disorder reported moderate to high levels of perceived social support. Overall, 62.86% of parents perceived high support, 33.57% perceived moderate support, and only 3.57% reported low support. Among the sources, significant others were the most supportive, with 76.43% of parents reporting high support. Family support was also strong, with 73.57% perceiving high support. In contrast, friends were a less consistent source, as only 47.14% reported high support, while a notable portion experienced moderate (38.57%) or low (14.29%) support. A study conducted in Selangor among the parents of children with ASD in autism care centers showed that 54.67% of the parents perceived high support from their family and friends, 33.33% perceived moderate support, and 8% perceived low support [53].

The predominance of family support in Nepal reflects deeply rooted cultural norms of joint and extended families, where caregiving is considered a collective duty rather than an individual one. However, this reliance on family may also leave parents vulnerable when relatives blame them or fail to accept the child’s condition. The weaker support from friends could be linked to stigma, as parents often avoid social gatherings to protect themselves and their children from negative remarks. Thus, social support in Nepal is both a protective factor (when families rally around the caregiver) and a risk factor (when stigma leads to blame or withdrawal of support).

In our study, parents emphasized the need for policies and government-led support initiatives for children with ASD and their families, similar to those available for other disabilities. Social support can be strengthened through government actions such as inclusive education policies, disability rights legislation, and targeted health programs. Parent training, improved access to specialized services, and inclusive education initiatives can help reduce parental stress, empower families, and promote greater societal acceptance. In our study, Physical/Material Well-being had the highest mean score among the five Family Quality of Life (FQoL) domains (mean/SD = 4.107/0.625), indicating that most families had relatively stable access to transportation, financial resources, and healthcare. Family Interaction followed closely with a mean of 4.092 (SD = 0.810), suggesting generally positive family relationships. In contrast, Emotional Well-being had the lowest mean score (3.61, SD = 0.800), highlighting that many parents faced emotional stress and challenges. A study conducted in Greece also found similar results as the mean FQoL score was highest in the physical/material wellbeing domain (mean/SD = 3.93/0.79), followed by family interaction (mean/SD = 3.85/0.75). Emotional well-being had the lowest FQoL mean score with a mean of 3.10 and a standard deviation of 0.82 [5]. A study in the Netherlands among parents of children with ASD reported the highest scores in the environmental QoL domain, which included financial resources, healthcare, and home environment [47]. Similarly, a study in Tamil Nadu, India found caregivers had better QoL in the environmental (38.3%) and social (35%) domains, reflecting stable finances, healthcare access, and social relations, while over half reported poor QoL in physical (56.6%) and psychological (59.1%) [14].

In Nepal, this imbalance may be explained by cultural dynamics: families often provide housing and financial support, buffering material and interaction domains, but the emotional burden remains high due to stigma, social isolation, and constant worry about the child’s future role in society. Unlike in Western contexts, where access to services may shape material well-being, Nepali families rely on their kinship networks for financial and caregiving support—yet emotional well-being lags behind due to cultural expectations and judgment from the wider community.

Our study found a significant negative correlation between parental stress and family quality of life among the parents (r = -0.903, p < 0.001). A study conducted in Greece also found a significant negative correlation with FQOL (p < 0.001) [5]. Similar findings have been shown by the studies conducted in other countries [27,54,55]. A qualitative study conducted in India found that families raising children with ASD have a low quality of life due to significant stress, which often results in social isolation and experiences of discrimination. The study highlighted that these families endure substantial economic and emotional challenges, leading to numerous unmet needs within the household [17].

In terms of coping strategies, our study found a significant negative correlation between both emotion-focused (r = –0.563, p < 0.001) and problem-focused (r = –0.845, p < 0.001) coping strategies and family quality of life, while no significant correlation was observed with avoidant coping. By contrast, a study conducted in Greece among mothers of children with ASD reported that family quality of life was negatively associated with the avoidant strategy “denial” (p < 0.05) but positively associated with emotion- and problem-focused strategies such as emotional support (p < 0.05), positive reframing (p < 0.05), and humour (p < 0.05) [5]. Similarly, a systematic review in Taiwan found that parents who used problem-focused strategies experienced stronger parent–child relationships and greater happiness [6].

The contrasting findings in Nepal may be explained by several contextual factors. Problem-focused coping often involves seeking therapies, planning interventions, and accessing professional services, but in Nepal, such services are scarce, costly, and largely concentrated in urban areas. Parents’ efforts to actively solve problems may therefore lead to frustration when resources are unavailable or outcomes are limited, which in turn reduces quality of life. Emotion-focused coping among Nepali parents often takes the form of resignation to fate, karma, or divine will, reflecting deep-rooted cultural and religious beliefs. While this acceptance may help reduce immediate stress, it can also discourage active help-seeking and contribute to ongoing emotional strain, thereby lowering quality of life. Stigma also plays a critical role: some parents cope emotionally by withdrawing from social interactions to avoid negative remarks, which protects them in the short term but reduces social support and undermines family well-being. In Nepali culture, mothers are primarily expected to shoulder caregiving responsibilities, often with limited external support. Parents who report higher use of coping strategies may also be those facing the greatest caregiving demands, social isolation, and uncertainty about their child’s future. In such cases, coping becomes a marker of burden rather than a pathway to improved outcomes.

Our study found a significant positive correlation between perceived social support and family quality of life among the parents (r = 0.685, p < 0.001). Studies conducted in Bangalore, India and Shanghai, China also showed that perceived social support had a significant positive correlation with quality of life among the parents of children with ASD [27,56].

Our study found that FQoL among parents of children with ASD was significantly related only to occupation, with unemployed parents reporting lower FQoL than homemakers (p = 0.003). No associations were observed with family income, education, employment status, religion, or age. In contrast, a study in Greece reported significant associations between mothers’ age, education, and employment with the FQoL subscale of physical/material well-being, while family income was linked to overall FQoL [5]. Similarly, research in Brazil identified family income and religion as predictors of FQoL among children with mild intellectual disability and ASD [57] and a study in Tamil Nadu, India found caregivers’ age significantly associated with physical, psychological, and social domains of quality of life [14]. This lack of significance underscores the complex interplay of social, cultural, and contextual factors shaping quality of life in Nepal, where determinants may differ from other settings.

A mixed-method study in Malaysia found that perceived stigma was negatively associated with resilience and quality of life, while resilience had a positive effect. Stigma, shaped by factors such as parents’ education, employment, child’s diagnosis age, and ASD severity, was shown to lower well-being, though resilient parents coped better [58,59]. Similarly, our study revealed that societal stigma led to social withdrawal and stress, but parents who managed stigma more effectively reported better quality of life. These parallels highlight how limited awareness, inadequate support, and cultural beliefs sustain stigma around ASD in both contexts.

This study used a mixed-method design to assess the quality of life, stress, and coping strategies of parents of children with ASD in Nepal, combining qualitative and quantitative approaches. The strengths include robust statistical methods and reliability measures like pretesting and inter-coder checks. However, some limitations should be noted. The use of purposive sampling and the subjective nature of participants’ experiences may limit the generalizability of the findings. The study was also limited to autism care centers in Kathmandu Valley, excluding parents who may not have access to such services. Therefore, the sample may overrepresent families with relatively better access to care and support, and the findings should be interpreted with caution regarding their generalizability to all parents of children with ASD in Nepal. The qualitative component included only mothers, which may introduce a gendered perspective and limits the representation of fathers’ experiences in the study. Despite these limitations, the findings provide important insights into parental stress, coping strategies, and family quality of life among parents of children with ASD in Nepal. This study highlights several areas for future research. Longitudinal studies could explore how parental stress, coping, and social support evolve over time and influence family well-being, while intervention-based research could assess the effectiveness of parental training, mental health support, and community-based inclusion programs. Further qualitative research may provide insights into cultural perceptions of ASD, stigma, and barriers to accessing services. Comparative studies across different regions and urban–rural settings in Nepal, along with perspectives from educators, healthcare providers, policymakers and autistic individuals could help inform more comprehensive and evidence-based policies and support programs for families.

## Conclusion

The study found that parental stress, particularly related to core autism symptoms, negatively affected family quality of life (FQoL). Emotion-focused and problem-focused coping strategies were also negatively correlated with FQoL, while perceived social support had a positive correlation. The parental occupation, and emotion-focused and problem focused coping strategies were identified as the significant predictors of the family quality of life after adjusting for the covariates.

Parents expressed concerns about their child’s future, particularly regarding communication and social development. They also faced societal stigma, which worsened their stress. The study highlighted challenges like physical and mental health issues, increased workload, and social isolation among the parents. Parents used coping strategies like emotional support and positive reframing, but schools and communities offered limited support. The lack of resources for children with ASD was another major challenge faced by the parents.

Combining both qualitative and quantitative data revealed that parental stress significantly impacts family quality of life. Acceptance, years of experience, and training participation were associated with better quality of life. The mixed-method approach highlighted the importance of societal stigma, coping mechanisms, and support systems in shaping parents’ well-being, emphasizing the need for better community support and policy improvements in Nepal.

Future research should focus on longitudinal studies, intervention-based programs, and qualitative insights into cultural perceptions of ASD in Nepal. Comparative studies across regions, examining stress in newly diagnosed parents, and involving educators, healthcare providers, and policymakers will help develop evidence-based policies and interventions to improve family well-being.

## Supporting information

S1 Tool Quantitative study tool (English). https://figshare.com/s/f30881dd6b2af07ac8a8.
(DOCX)

S2 Tool Qualitative interview guide (English). https://figshare.com/s/c48120617a7e48f023b1.
(DOCX)

S1 Checklist MMAT reporting checklist [60,61]. https://figshare.com/s/ad8d77094920c71685b4.
(DOCX)
