# Correction: Quality of life, stress and its coping strategies among the parents of children with autism spectrum disorder in Kathmandu Valley: A mixed method study

**DOI:** 10.1371/journal.pgph.0007504

**Published:** 2026-10-16

**Authors:** Sajani Basan, Amit Arjyal, Shital Bhandary

The corresponding author’s email address is incorrect. Sajani Basan’s email address is: sajani.basan@gmail.com.
