# Supplementary material for: Quality of life, stress and its coping strategies among the parents of children with autism spectrum disorder in Kathmandu Valley: A mixed method study
Source: PLOS Glob Public Health. 2026 Aug 21;6(8):e0006997. doi: 10.1371/journal.pgph.0006997 (PMC13496449; doi:10.1371/journal.pgph.0006997)
Supplement: Supplementary file 1 [file pgph.0006997.s001.docx]

**QUANTITATIVE TOOL**

A. **Socio-demographic Information**

| Date of interview: | | |
| --- | --- | --- |
| Participant Code: | | |
| Address of respondent: | | |
| **Information about the child with ASD** | | |
| 1. Age of diagnosis | | …..Years …..Months |
| 2. Age (completed years) | |  |
| 3. Sex | | 1. Male 2. Female |
| **Information about parent** | | |
| 1. Age (completed years) |  | |
| 2. Sex | 1. Male 2. Female | |
| 3. Relation to the child diagnosed with ASD | 1. Father 2. Mother 3. Grandparent 4. Others | |
| 4. Highest level of education | 1. No education 2. Basic level education (1-8) 3. Secondary level of education (9-12) 4. Bachelor’s level and above | |
| 5. Main occupation of respondent | 1. Agriculture 2. Homemaker 3. Job/service 4. Labor 5. Business/commerce 6. Unemployed 7. Others | |
| 6. Income status of family | 1. ≤ 4850 2. 4851-14550 3. 14551-24350 4. 24351-36550 5. 36551-48750 6. 48751-97450 7. ≥ 97451 | |
| 7. Education of household head | 1. Professional or honors 2. Graduate or postgraduate 3. Intermediate of post high school diploma 4. High school certificate 5. Middle school certificate 6. Primary school or literate 7. Illiterate | |
| 8. Main occupation of household head | 1. Profession 2. Semi profession 3. Clerical, shop owner, farmer 4. Skilled worker 5. Semi-skilled worker 6. Unskilled worker 7. Unemployment | |
| 9. Family Type | 1.Nuclear  2. Joint | |
| 10. Ethnicity | 1. Dalit 2. Janajati 3. Madhesi 4. Muslim 5. Brahmin/Chhetri 6. Others | |
| 11. Religion | 1. Hindu 2. Buddhist 3. Islam 4. Kirat 5. Christian 6. Others | |

B. **Parental Stress**

|  | **Not stressful** | **Sometimes creates stress** | **Often creates stress** | **Very stressful on a daily basis** | **So stressful sometimes we feel we can’t cope** |
| --- | --- | --- | --- | --- | --- |
| 1. Your child’s social development |  |  |  |  |  |
| 2. Your child’s ability to communicate |  |  |  |  |  |
| 3. Tantrums/meltdowns |  |  |  |  |  |
| 4. Aggressive behavior (siblings, peers) |  |  |  |  |  |
| 5. Self-injurious behavior |  |  |  |  |  |
| 6. Difficulty making transitions from one activity to another |  |  |  |  |  |
| 7. Sleep problems |  |  |  |  |  |
| 8. Your child’s diet |  |  |  |  |  |
| 9. Bowel problems (diarrhea, constipation) |  |  |  |  |  |
| 10. Potty training |  |  |  |  |  |
| 11. Not feeling close to your child |  |  |  |  |  |
| 12. Concern for the future of your child being accepted by others |  |  |  |  |  |
| 13. Concern for the future of your child living independently |  |  |  |  |  |

C. **Coping Strategies**

|  |  | **I haven’t been doing this at all** | **A little bit** | **A medium amount** | **I’ve been doing this a lot** |
| --- | --- | --- | --- | --- | --- |
| 1 | I've been turning to work or other activities to take my mind off things. |  |  |  |  |
| 2 | I've been concentrating my efforts on doing something about the situation I'm in. |  |  |  |  |
| 3 | I've been saying to myself "this isn't real". |  |  |  |  |
| 4 | I've been using alcohol or other drugs to make myself feel better |  |  |  |  |
| 5 | I've been getting emotional support from others. |  |  |  |  |
| 6 | I've been giving up trying to deal with it. |  |  |  |  |
| 7 | I've been taking action to try to make the situation better. |  |  |  |  |
| 8 | I've been refusing to believe that it has happened. |  |  |  |  |
| 9 | I've been saying things to let my unpleasant feelings escape. |  |  |  |  |
| 10 | I’ve been getting help and advice from other people. |  |  |  |  |
| 11 | I've been using alcohol or other drugs to help me get through it. |  |  |  |  |
| 12 | I've been trying to see it in a different light, to make it seem more positive. |  |  |  |  |
| 13 | I’ve been criticizing myself. |  |  |  |  |
| 14 | I've been trying to come up with a strategy about what to do. |  |  |  |  |
| 15 | I've been getting comfort and understanding from someone. |  |  |  |  |
| 16 | I've been giving up the attempt to cope. |  |  |  |  |
| 17 | I've been looking for something good in what is happening. |  |  |  |  |
| 18 | I've been making jokes about it. |  |  |  |  |
| 19 | I've been doing something to think about it less, such as going to movies, watching TV, reading, daydreaming, sleeping, or shopping. |  |  |  |  |
| 20 | I've been accepting the reality of the fact that it has happened. |  |  |  |  |
| 21 | I've been expressing my negative feelings. |  |  |  |  |
| 22 | I've been trying to find comfort in my religion or spiritual beliefs. |  |  |  |  |
| 23 | I’ve been trying to get advice or help from other people about what to do. |  |  |  |  |
| 24 | I've been learning to live with it. |  |  |  |  |
| 25 | I've been thinking hard about what steps to take. |  |  |  |  |
| 26 | I’ve been blaming myself for things that happened |  |  |  |  |
| 27 | I've been praying or meditating |  |  |  |  |
| 28 | I've been making fun of the situation. |  |  |  |  |

D. **Perceived Social Support**

|  | **Very strongly disagree** | **Strongly disagree** | **Mildly**  **disagree** | **Neutral** | **Mildly agree** | **Strongly agree** | **Very strongly agree** |
| --- | --- | --- | --- | --- | --- | --- | --- |
| There is a special person who is around when I am in need. |  |  |  |  |  |  |  |
| There is a special person with whom I can share joys and sorrows |  |  |  |  |  |  |  |
| My family really tries to help me |  |  |  |  |  |  |  |
| I get the emotional help and support I need from my family |  |  |  |  |  |  |  |
| I have a special person who is a real source of comfort to me |  |  |  |  |  |  |  |
| My friends really try to help me |  |  |  |  |  |  |  |
| I can count on my friends when things go wrong |  |  |  |  |  |  |  |
| I can talk about my problems with my family |  |  |  |  |  |  |  |
| I have friends with whom I can share my joys and sorrows |  |  |  |  |  |  |  |
| There is special person in my life who cares about my feelings |  |  |  |  |  |  |  |
| My family is willing to help me make decisions |  |  |  |  |  |  |  |
| I can talk about my problems with my friends |  |  |  |  |  |  |  |

E. **Family Quality of Life**

| **How satisfied am I that…** | **Very Dissatisfied** | **Dissatisfied** | **Neither** | **Satisfied** | **Very Satisfied** |
| --- | --- | --- | --- | --- | --- |
| 1. My family enjoys spending time together. |  |  |  |  |  |
| 2. My family members help the children learn to be independent. |  |  |  |  |  |
| 3. My family has the support we need to relieve stress. |  |  |  |  |  |
| 4. My family members have friends or others who provide support. |  |  |  |  |  |
| 5. My family members help the children with schoolwork and activities. |  |  |  |  |  |
| 6. My family members have transportation to get to the places they need to be. |  |  |  |  |  |
| 7. My family members talk openly with each other. |  |  |  |  |  |
| 8. My family members teach the children how to get along with others. |  |  |  |  |  |
| 9. My family members have some time to pursue our own interests. |  |  |  |  |  |
| 10. Our family solves problems together. |  |  |  |  |  |
| 11. My family members support each other to accomplish goals. |  |  |  |  |  |
| 12. My family members show that they love and care for each other. |  |  |  |  |  |
| 13. My family has outside help available to us to take care of special needs of all family members. |  |  |  |  |  |
| 14. Adults in our family teach the children to make good decisions. |  |  |  |  |  |
| 15. My family gets medical care when needed. |  |  |  |  |  |
| 16. My family has a way to take care of our expenses. |  |  |  |  |  |
| 17. Adults in my family know other people in the children’s lives (friends, teachers, etc.). |  |  |  |  |  |
| 18. My family is able to handle life’s ups and downs. |  |  |  |  |  |
| 19. Adults in my family have time to take care of the individual needs of every child. |  |  |  |  |  |
| 20. My family gets dental care when needed. |  |  |  |  |  |
| 21. My family feels safe at home, work, school, and in our neighborhood. |  |  |  |  |  |
| 22. My family member with a disability has support to accomplish goals at school or at workplace. |  |  |  |  |  |
| 23. My family member with a disability has support to accomplish goals at home. |  |  |  |  |  |
| 24. My family member with a disability has support to make friends. |  |  |  |  |  |
| 25. My family has good relationships with the service providers who provide services and support to our family member with a disability. |  |  |  |  |  |
