# Supplementary material for: Quality of life, stress and its coping strategies among the parents of children with autism spectrum disorder in Kathmandu Valley: A mixed method study
Source: PLOS Glob Public Health. 2026 Aug 21;6(8):e0006997. doi: 10.1371/journal.pgph.0006997 (PMC13496449; doi:10.1371/journal.pgph.0006997)
Supplement: Supplementary file 2 [file pgph.0006997.s002.docx]

**QUALITATIVE TOOL**

The socio-demographic information about the respondents will be taken from the quantitative interviews based on participant code.

**Parental stress, coping strategies and perceived social support**

| **Question** | **Probes** |
| --- | --- |
| After your child was diagnosed with ASD, how has it affected your life? | In terms of daily routine, time management (care of child, other regular works), work life, work load, financial burden, physical and emotional condition, others |
| How is the current situation of your child? What kind of challenges does your child face because of ASD? | Child’s social development, ability to communicate, sleep problems, behavior (tantrums, aggressive behavior, self-injurious behavior, eating habits and others) |
| How does your child’s current conditions and behavior affect your health? | Physical health (rest, sleep, fatigue, pain), psychological health (Stress, fear, anger, worry), social (social relations, social interaction, support), environmental (family environment) |
| What concerns do you have about your child having ASD? (Types of stress) | Stress related to child’s behavior, child’s development, child’s future (Child being accepted by others, child living independently in the future), Stress related to child-parent relationship and interaction |
| How do you cope with the challenges and stress you face while raising your child? | Self-distraction, active coping, substance use, denial, planning, self-blame, emotional support, informational support, venting…. |
| How do you perceive the support you get from your family while raising your child? | Help in raising child, emotional support, sharing feelings |
| How do you perceive the support you get from your friends while raising your child? | Emotional support, sharing feelings |
| How do you perceive the support you get from others around you while raising your child? | Neighbors, relatives |
| What kind of interventions do you think can help parents like you who have children with ASD? |  |

**Family Quality of Life**

| **Question** | **Probes** |
| --- | --- |
| How is the family relationships and interactions in your family? | Spending time together, interaction with each other, problem solving, supporting each other, showing love and care to each other, able to handle life’s ups and downs |
| How do you feel about the support and resources available to you in your role as a parent? (How do the family members in your family engage in parenting of your child?) | Help children learn to be independent, help them with schoolwork or their activities, help them get along with others, teach children make good decisions, know other people in the children’s lives… |
| How is the status of your family’s emotional wellbeing? How does your family cope with stress and emotional challenges? | Support in relieving stress, support from friends and others, have time to pursue their own interests |
| How does your family manage its physical and material needs? | Transportation, health care, financial stability, safety |
| What kinds of support does your child receive in your family? How well does your family support the child with ASD? | Family’s support and relationship with the child with ASD |
