# Supplementary material for: Quality of life, stress and its coping strategies among the parents of children with autism spectrum disorder in Kathmandu Valley: A mixed method study
Source: PLOS Glob Public Health. 2026 Aug 21;6(8):e0006997. doi: 10.1371/journal.pgph.0006997 (PMC13496449; doi:10.1371/journal.pgph.0006997)
Supplement: Supplementary file 3 [file pgph.0006997.s003.docx]

**Reporting Guideline for The Study: Mixed Methods Appraisal Tool (MMAT), version 2018**

**Part I: Checklist**

| **Category of study designs** | **Methodological quality criteria** | **Responses** | | | |
| --- | --- | --- | --- | --- | --- |
|  |  | **Yes** | **No** | **Can’t tell** | **Comments** |
| Screening questions (for all types) | S1. Are there clear research questions? | Yes |  |  |  |
|  | S2. Do the collected data allow to address the research questions? | Yes |  |  |  |
|  | *Further appraisal may not be feasible or appropriate when the answer is ‘No’ or ‘Can’t tell’ to one or both screening questions.* | | | | |
| 1. Qualitative | 1.1. Is the qualitative approach appropriate to answer the research question? |  |  |  |  |
|  | 1.2. Are the qualitative data collection methods adequate to address the research question? |  |  |  |  |
|  | 1.3. Are the findings adequately derived from the data? |  |  |  |  |
|  | 1.4. Is the interpretation of results sufficiently substantiated by data? |  |  |  |  |
|  | 1.5. Is there coherence between qualitative data sources, collection, analysis and interpretation? |  |  |  |  |
| 2. Quantitative randomized controlled trials | 2.1. Is randomization appropriately performed? |  |  |  |  |
|  | 2.2. Are the groups comparable at baseline? |  |  |  |  |
|  | 2.3. Are there complete outcome data? |  |  |  |  |
|  | 2.4. Are outcome assessors blinded to the intervention provided? |  |  |  |  |
|  | 2.5 Did the participants adhere to the assigned intervention? |  |  |  |  |
| 3. Quantitative non- randomized | 3.1. Are the participants representative of the target population? |  |  |  |  |
|  | 3.2. Are measurements appropriate regarding both the outcome and intervention (or exposure)? |  |  |  |  |
|  | 3.3. Are there complete outcome data? |  |  |  |  |
|  | 3.4. Are the confounders accounted for in the design and analysis? |  |  |  |  |
|  | 3.5. During the study period, is the intervention administered (or exposure occurred) as intended? |  |  |  |  |
| 4.Quantitative descriptive | 4.1. Is the sampling strategy relevant to address the research question? |  |  |  |  |
|  | 4.2. Is the sample representative of the target population? |  |  |  |  |
|  | 4.3. Are the measurements appropriate? |  |  |  |  |
|  | 4.4. Is the risk of nonresponse bias low? |  |  |  |  |
|  | 4.5. Is the statistical analysis appropriate to answer the research question? |  |  |  |  |
| 5. Mixed methods | 5.1. Is there an adequate rationale for using a mixed methods design to address the research question? | Yes |  |  |  |
|  | 5.2. Are the different components of the study effectively integrated to answer the research question? | Yes |  |  |  |
|  | 5.3. Are the outputs of the integration of qualitative and quantitative components adequately interpreted? | Yes |  |  |  |
|  | 5.4. Are divergences and inconsistencies between quantitative and qualitative results adequately addressed? | Yes |  |  |  |
|  | 5.5. Do the different components of the study adhere to the quality criteria of each tradition of the methods involved? | Yes |  |  |  |

**Part II: Explanations**

| **5. Mixed methods studies** | **Methodological quality criteria** |
| --- | --- |
| Mixed methods (MM) research involves combining qualitative (QUAL) and quantitative (QUAN) methods. In this tool, to be considered MM, studies have to meet the following criteria (62): (a) at least one QUAL method and one QUAN method are combined; (b) each method is used rigorously in accordance to the generally accepted criteria in the area (or tradition) of research invoked; and (c) the combination of the methods is carried out at the minimum through a MM design (defined *a priori*, or emerging) and the integration of the QUAL and QUAN phases, results, and data.  Common designs include (this list if not exhaustive):  **Convergent design**  The QUAL and QUAN components are usually (but not necessarily) concomitant. The purpose is to examine the same phenomenon by interpreting QUAL and QUAN results (bringing data analysis together at the interpretation stage), or by integrating QUAL and QUAN datasets (e.g., data on same cases), or by transforming data (e.g., quantization of qualitative data).  **Sequential explanatory design**  Results of the phase 1 - QUAN component inform the phase 2 - QUAL component. The purpose is to explain QUAN results using QUAL findings. E.g., the QUAN results guide the selection of QUAL data sources and data collection, and the QUAL findings contribute to the interpretation of QUAN results.  **Sequential exploratory design**  Results of the phase 1 - QUAL component inform the phase 2 - QUAN component. The purpose is to explore, develop and test an instrument (or taxonomy), or a conceptual framework (or theoretical model). E.g., the QUAL findings inform the QUAN data collection, and the QUAN results allow a statistical generalization of the QUAL findings. | Our study is a cross-sectional study with convergent mixed method design. Quantitative and qualitative data were collected concurrently, analyzed independently, and subsequently integrated during the interpretation phase. Quantitative data were obtained from 140 parents, while qualitative data were collected from a purposively selected subset of 11 parents drawn from the quantitative sample, based on their responses and key characteristics to ensure inclusiveness. |
|  | 5.1. Is there an adequate rationale for using a mixed methods design to address the research question?  Explanations:  An adequate rationale for using a mixed methods design is provided in this study. Parental stress, coping strategies, and quality of life in the context of caring for a child with autism spectrum disorder are complex and inherently subjective experiences that cannot be fully understood through quantitative measurement alone. While the quantitative component allows for the assessment of the level and distribution of stress, coping strategies, and quality of life among parents, it is limited in capturing the personal meanings, contextual influences, and lived experiences underlying these outcomes.  The qualitative component complements and enriches the quantitative findings by exploring parents’ individual experiences, perceptions, and narratives. Parents differ in their family dynamics, social support, cultural context, and personal coping capacities, all of which influence mental health and quality of life. Integrating qualitative insights with quantitative results therefore provides a more comprehensive and nuanced understanding of the phenomenon. Overall, the convergent mixed methods design strengthens the study by producing a detailed and realistic portrayal of parental experiences. |
|  | 5.2. Are the different components of the study effectively integrated to answer the research question?  Explanations  The different components of the study were effectively integrated to address the research question. Quantitative and qualitative data were collected concurrently as part of a convergent mixed methods design. During the quantitative data collection phase, parents who demonstrated potential for in-depth exploration were identified, and qualitative interviews were subsequently conducted with their informed consent at times convenient to the participants.  Quantitative and qualitative data were analyzed independently using appropriate analytic procedures for each component. Integration occurred at the interpretation stage through systematic triangulation of findings from both datasets. The results were brought together to develop a comprehensive understanding of parental stress, coping strategies, and quality of life. The integrated findings were organized and presented as convergent findings (where qualitative and quantitative results corroborated each other), divergent findings (where results differed or contradicted), and expansive findings (where qualitative data extended or elaborated on quantitative results). This explicit integration of the two components ensured that the mixed methods design effectively addressed the research question. |
|  | 5.3. Are the outputs of the integration of qualitative and quantitative components adequately interpreted?  Explanations  The outputs of the integration of the qualitative and quantitative components were adequately interpreted through meta-inference. Integrating findings from both components added substantial value to the study by enabling a deeper and more comprehensive understanding of parents’ experiences of caring for a child with autism spectrum disorder. While the quantitative component quantified levels of stress, coping strategies, social support, and quality of life and identified associated factors, it could not fully explain the underlying meanings, processes, and contextual influences shaping these outcomes.  The qualitative component not only helped explain and contextualize the quantitative results but also generated expansive findings that could not be captured through standardized quantitative measures. These included nuanced insights into parents’ emotional journeys following diagnosis, family and societal dynamics, stigma-related experiences, and individual coping processes. Through meta-inference, the integrated interpretation demonstrated how quantitative patterns were corroborated by qualitative narratives, where findings diverged, and how qualitative data expanded the overall understanding beyond what was measurable quantitatively. This comprehensive interpretation highlights the added value of the mixed methods design by providing a detailed and realistic portrayal of how multiple factors interact to influence parental stress, coping, and quality of life. |
|  | 5.4. Are divergences and inconsistencies between quantitative and qualitative results adequately addressed?  Explanations  Divergences and inconsistencies between the quantitative and qualitative results were adequately addressed in this study. During the integration phase, areas of divergence between the two components were explicitly identified and examined. Rather than merely reporting these discrepancies, the study provided plausible explanations for the divergent findings by considering contextual, methodological, and experiential factors influencing parents’ responses. Potential causes such as differences in individual circumstances, social and family contexts, and variations in how experiences were captured through standardized measures versus in-depth interviews were discussed. This interpretive approach helped reconcile inconsistencies and strengthened the overall understanding of the findings. |
|  | 5.5. Do the different components of the study adhere to the quality criteria of each tradition of the methods involved?  Explanations  **1. Qualitative Component:**  The qualitative approach was appropriate for exploring the parents’ lived experiences, perceptions, and subjective meanings related to stress, coping strategies, and quality of life while caring for a child with autism spectrum disorder. An in-depth qualitative approach allowed for exploration of complex psychosocial processes that could not be adequately captured through quantitative methods alone.  Qualitative data were collected using in-depth interviews, which are well suited to exploring sensitive topics such as emotional experiences, family dynamics, social support, and coping. Before data collection, written informed consent was obtained from the parents. Interviews were conducted at times convenient for participants, ensuring comfort and openness. The data collection method was appropriate to generate rich, detailed accounts relevant to the research objectives.  The trustworthiness of the qualitative tool and procedures were maintained as different measures were applied to ensure credibility, dependability, transferability and confirmability, and was properly explained too.  Qualitative findings were systematically derived from the data using Braun and Clark’s steps of thematic analysis of the data. Themes were developed through careful examination of interview transcripts, ensuring that findings were grounded in participants’ narratives rather than preconceived assumptions.  Interpretations of qualitative findings were supported by direct evidence from the data, including illustrative participant quotations. These quotes adequately represented the themes and strengthened the credibility and trustworthiness of the interpretations.  There was clear coherence across qualitative data sources, methods of data collection, analytical procedures, and interpretation. The alignment between the research question, interview approach, thematic analysis, and interpretation ensured methodological consistency and rigor.  **2. Quantitative Component**  The sampling strategy was appropriate for addressing the research question, as the study targeted parents of children diagnosed with autism spectrum disorder, the population of interest. Given the sensitive nature of autism and the associated stigma, identifying and recruiting an adequate number of participants posed practical challenges. Therefore, a non-probability purposive sampling technique was employed to select study participants. Autism care centers were identified using a snowball sampling approach. Purposive sampling was considered suitable because parents of children with ASD constitute a hard-to-reach population and may be reluctant to participate. This approach enabled us to directly engage parents who were most likely to provide relevant and meaningful information on stress, coping strategies, social support, and quality of life.  The sample adequately represented the target population, with clear inclusion and exclusion criteria. The characteristics of the participants were described in sufficient detail, allowing assessment of the sample’s relevance to parents of children with autism spectrum disorder within the study context.  Quantitative measurements were appropriate and relevant to the research objectives. Standardized, validated, and reliability-tested instruments were used to measure parental stress, coping strategies, social support, and quality of life. These measures were suitable for accurately capturing the constructs of interest.  The risk of nonresponse bias was low. The purposive sampling strategy helped minimize potential nonresponse, which could have been higher given the sensitive nature of the study population. The response rate was satisfactory, and conducting data collection through direct interviews further reduced the likelihood of nonresponse.  The statistical analyses were appropriate for the cross-sectional design and aligned with the research questions. Descriptive and inferential analyses were clearly described, justified, and suitable for examining associations between stress, coping strategies, social support, and quality of life.  Both the qualitative and quantitative components of the study adhered to the quality criteria of their respective methodological traditions. The qualitative component demonstrated coherence, and trustworthiness, while the quantitative component showed methodological rigor through validity and reliability, valid measurements, low risk of bias, and suitable statistical analysis. As neither component exhibited major methodological weaknesses, the overall mixed methods study can be considered of high quality. The integration of two high-quality components strengthened the validity of the findings and ensured that the overall quality of the mixed methods study was not compromised by its weakest component. |
